# Evaluation of obstructive sleep apnea in non-cystic fibrosis bronchiectasis: A cross-sectional study

**DOI:** 10.1371/journal.pone.0185413

**Published:** 2017-10-03

**Authors:** Newton Santos Faria Júnior, Jessica Julioti Urbano, Israel Reis Santos, Anderson Soares Silva, Eduardo Araújo Perez, Ângela Honda Souza, Oliver Augusto Nascimento, José Roberto Jardim, Giuseppe Insalaco, Luis Vicente Franco Oliveira, Roberto Stirbulov

**Affiliations:** 1 Master’s degree and PhD Program in Surgery Research, Santa Casa de Sao Paulo School of Medical Sciences, (FCMSCSP), Sao Paulo (SP), Brazil; 2 Rehabilitation Sciences Master’s degree and PhD Program, Nove de Julho University (UNINOVE), Sao Paulo (SP), Brazil; 3 Pulmonary Rehabilitation Center, Federal University of Sao Paulo, (UNIFESP), Sao Paulo (SP), Brazil; 4 National Research Council of Italy–Institute of Biomedicine and Molecular Immunology “Alberto Monroy”, Palermo (SI), Italy; 5 Medicine School, University Center of Anapolis (UniEVANGELICA), Anapolis (GO), Brazil; University of Rome Tor Vergata, ITALY

## Abstract

The relationship between sleep disorders and bronchiectasis has not been well described. We hypothesize that, due to the irreversible dilatation of the bronchi, the presence of secretions, and airflow obstruction, patients with non-cystic fibrosis bronchiectasis may be predisposed to hypoxemia during sleep, or to symptoms that may lead to arousal. A cross-sectional observational study was performed involving 49 patients with a clinical diagnosis of non-cystic fibrosis bronchiectasis (NCFB). All patients underwent clinical evaluation, spirometry, and polysomnography, and were evaluated for the presence of excessive daytime sleepiness (EDS) and risk of obstructive sleep apnea (OSA). The mean age of the participants was 50.3 ± 13.6 years; 51.1% of patients were male and had a mean body mass index of 23.8 ± 3.4 kg/m^2^. The mean total sleep time (TST) was 325.15 ± 64.22 min with a slight reduction in sleep efficiency (84.01 ± 29.2%). Regarding sleep stages, stage 1 sleep and REM sleep were abnormal. OSA was present in 40.82% of the patients. The mean arousal index was 5.6 ± 2.9/h and snoring was observed in 71.43% of the patients. The oxygen desaturation index (ODI) was 14.35 ± 15.36/h, mean minimum oxygen saturation (SpO_2_ nadir) was 83.29 ± 7.99%, and mean TST with an SpO_2_ less than 90% was 30.21 ± 60.48 min. EDS was exhibited by 53.06% of the patients and 55.1% were at high risk of developing OSA. The patients infected by *Pseudomonas aeruginosa* had higher apnea-hypopnea indices, ODI, and TST with SpO_2_ < 90%, and lower values of SpO_2_ nadir. Adult patients with clinically stable NCFB, especially those infected by *Pseudomonas aeruginosa*, display EDS and a high prevalence of OSA, associated with considerable oxygen desaturation during sleep.

## Introduction

Bronchiectasis (BCTS) is a chronic disease characterized by irreversible, permanent, and abnormal dilatation of the bronchi and bronchioles [1–5]. The primary cause of this condition is repeated cycles of infection and inflammation, leading to progressive destruction of the airways, reduced mucociliary clearance, excessive production of sputum, and a progressive decline in lung function [6–9].

The prevalence of BCTS is not known definitively; it probably varies significantly among different populations [10]. It is estimated that there are in the United States of America at least 110,000 adult patients diagnosed with BCTS: 4.2 per 100,000 people between 18 and 34 years of age, and 272 per 100,000 people aged 75 years or older [11]. An epidemiological study from Finland suggests an incidence of 2.7 per 100,000 people [12], while in New Zealand, an overall incidence of 3.7 per 100,000 children was noted [13]. Certain demographic groups, such as those with little access to health, lower socio-economic status, and high rates of lung infection in childhood, are at high risk for BCTS [14–15]. In a study of 42,500 admissions to a Brazilian hospital specializing in lung diseases, 0.4% of patients (170) hospitalized between 1978 and 2001 were diagnosed with BCTS [16]. Another recent study conducted in Germany found an average annual rate of hospital admissions of patients with BCTS of 9.4 per 100,000 [17].

Sleep disorders have a high prevalence in the general population: they are now considered an important public health problem, affecting about 45% of the world’s population [18]. A study published in 1993 showed that the prevalence of obstructive sleep apnea (OSA) varied from 2% to 3% in women and 4% to 5% in men [19]. In young adults in the Western world, OSA affects 3% to 7% of the male population and 2.5% of females [20]. A survey of a representative population of the city of Sao Paulo showed that 24.8% of men and 9.6% of women had OSA [21]. In patients with chronic obstructive pulmonary disease (COPD), the prevalence of OSA is 9.5% to 14% [22–24]. Among patients with cystic fibrosis (CF), about 3.9% meet the criteria for OSA [25]. Among patients with asthma, the incidence of OSA was 2.51 times greater than that in the control group [26]. In another study of patients with COPD, 24.7% experienced excessive daytime sleepiness [27].

The research literature has linked certain respiratory diseases with sleep disorders [27–31]; however, the relationship between sleep disorders and BCTS has not been well described. Recently, two studies that investigated the presence of sleep disorders in patients with BCTS [32–33] have been published. Both assessed the quality of sleep through specific questionnaires; however, neither study used standard overnight polysomnography (PSG), considered the gold standard for evaluating the presence of sleep disorders.

To our knowledge, this is the first study to describe the physiological sleep patterns in patients with non-cystic fibrosis bronchiectasis (NCFB), using PSG. We believe that this study may contribute to a better understanding of the clinical course of the disease and lead to potential therapeutic interventions.

We hypothesize that, due to the irreversible dilatation of the bronchi, the presence of sputum, and airflow obstruction, patients with NCFB may be predisposed to hypoxemia during sleep, or to symptoms that may lead to arousal. The objective of the present study was to describe physiological variables of sleep in patients with NCFB through PSG, and to stratify these patients by the risk of OSA and excessive daytime sleepiness (EDS).

## Materials and methods

### Study design and ethical considerations

A cross-sectional observational study was performed at the Sleep Laboratory in Sao Paulo, Brazil. The patients were recruited from two BCTS Outpatient Clinics in Sao Paulo, Brazil, between April 2013 and March 2016. The study protocol is being published elsewhere [34].

**Fig 1:**
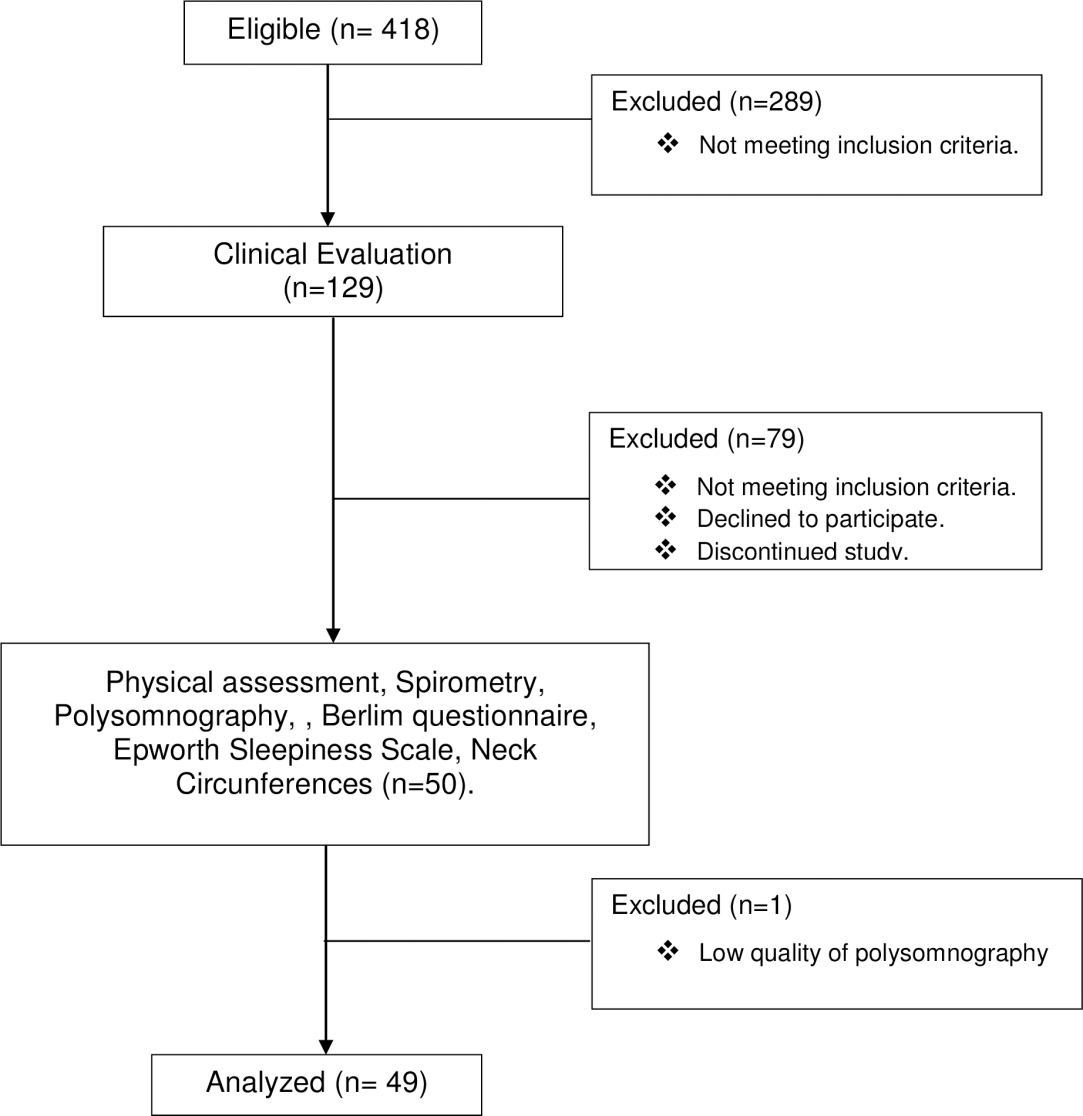
Flowchart of the study design.

The design, conduct, and reporting of this study followed the norms of the Strengthening the Reporting of Observational Studies in Epidemiology (STROBE) statement [35]; they were compliant with the ethical standards established in the 1961 Declaration of Helsinki (as revised in Hong Kong in 1989, and in Edinburgh, Scotland in 2000) and with the Regulatory Guidelines and Norms for Research Involving Human Subjects of the National Health Board of the Brazilian Health Ministry, according to Resolution 466/2012. Fig 1 shows the flowchart of the study.

This study was approved by the Human Research Ethics Committees of Santa Casa de Misericordia Hospital (Sao Paulo, Brazil; process number 178/2012). All participants signed an informed consent form. They were allowed to leave the study at any time without negative consequences.

### Eligibility criteria

The patient cohort comprised those diagnosed with BCTS based on the results of high-resolution chest computerized tomography, which is the gold standard for detecting this disease [36–37]. Other inclusion criteria were age between 18 and 65 years, use of a long-acting bronchodilator (BD), clinical stability for at least one month, and willingness to participate in the study and provide signed informed consent. Exclusion criteria were BCTS stemming from CF (i.e., chloride level in sweat > 60 mmol/L), a history of smoking, other lung diseases such as COPD, asthma and/or other comorbidities that may affect the diagnosis and/or prognosis of BCTS, or inability of the patient to understand the administered questionnaire.

Patients who had exacerbations of their clinical condition during the medical anamnesis were eligible to be included in the study if they were clinically stable for one month prior to their next physician's appointment; these patients participated in the study at their three-month follow-up appointment.

### Clinical evaluation

Initially, 418 eligible patients with clinical records and confirmed diagnosis of BCTS were recruited at two reference centers for respiratory diseases in the city of Sao Paulo. Of these, 289 patients who did not meet the inclusion criteria were excluded. Information regarding the symptoms-specifically, the presence of cough, sputum, hemoptysis, dyspnea, fatigue, wheezing, and rhinosinusitis, the number of exacerbations per year, the drugs administered, comorbidities, and etiology of the disease-were collected from 129 medical records. After the data collection, the clinical evaluation and the analysis of sputum samples for the presence of Pseudomonas aeruginosa (PA) in the clinical laboratories attached to the services involved in the study were performed.

After this step, 79 patients were excluded because they did not fully meet the inclusion criteria, decided not to participate in the study or discontinued the protocol. The remaining 50 patients were referred to the sleep laboratory to perform the physical assessment with the BMI [38] and neck circumference (NC) [39], pulmonary function tests, PSG overnight tests and respond to the Berlin questionnaire and The Epworth Sleepiness Scale. These questionnaires were administered during the morning following PSG exams. Therefore, the presence of snoring and excessive daytime sleepiness were not used as inclusion criteria. The final analysis was performed with 49 patients due to the exclusion of a patient who presented a PSG with poor technical quality.

The Berlin Questionnaire was applied to determine the risk of OSA [40]. This questionnaire has 10 items organized into three categories: apnea and snoring, daytime sleepiness, and systemic arterial hypertension and obesity. The patients were divided into low risk or high risk of OSA. To verify the presence of EDS, the simple and self-administered Portuguese language version of the Epworth Sleepiness Scale (ESS) questionnaire was used [41–43]. This questionnaire addresses situations involving the occurrence of daytime sleepiness during specific activities of daily living, in adults, by rating their likelihood of experiencing the desire to sleep or nap, in eight situations, on a scale from 0 to 3.

### Polysomnography

All patients underwent PSG employing the system Somnologica Studio–Embla A10 version 3.1.2 (Medcare Flaga Hs. Medical Devices, Reykjavik, Iceland). The interpretation of the results was based on the guidelines of the American Academy of Sleep Medicine (AASM). Patients with an apnea-hypopnea index (AHI) of ≥ 5 events per hour of total sleep time were classified as having OSA. The AHI was calculated as the number of apneas and hypopneas per hour of total sleep time. OSA was defined as a lack of airflow, or a reduction of ≥ 90% in the airflow signal for at least 10 s, coupled with signs/symptoms (eg, associated sleepiness, fatigue, insomnia, snoring, subjective nocturnal respiratory disturbance, or observed apnea) or associated medical or psychiatric disorder (ie, hypertension, coronary artery disease, atrial fibrillation, congestive heart failure, stroke, diabetes, cognitive dysfunction, or mood disorder). Obstructive Hypopnea was defined as a discernible drop in airflow of ≥ 30% with respect to the baseline, for at least 10 s, followed by a peripheral oxyhemoglobin desaturation of ≥ 4% [44–46].

### Spirometry

All patients underwent spirometry in accordance with the guidelines of the American Thoracic Society and the European Respiratory Society [47]. Forced vital capacity (FVC), forced expiratory volume in the first second (FEV_1_), and FEV_1_/FVC ratio were measured before and after the use of a short-acting bronchodilator [48]. The spirometry tests were performed using the KoKo PFT system, version 4.11 (nSpire Health, Inc., Longmont, CO, USA).

### Data analysis

#### Sample size

Because of the paucity of data in the literature regarding the evaluation of sleep disorders in adult patients with NCFB, a pilot study was carried out to calculate the appropriate sample size using a prevalence rate of 0.238 for OSA with 90% confidence level, and a 20% error (i.e., ± 10%). For this study, we determined that a sample size of 49 patients was required.

#### Statistical analyses

The Kolmogorov-Smirnov test was carried out first, to determine the presence or absence of data normality. The data was represented by mean±standard deviation of normally distributed or median (interquartile interval) for not normally distributed data. The spirometry values pre-BD and post-BD were compared using the Student *t* test for paired samples. For comparisons the presence of PA, azithromycin therapy and exacerbations ≥ 3 times a week and PSG variables between individuals with and without OSA, we used, the Student *t* test or the nonparametric Mann-Whitney *U* test, for quantitative variables. When the variables were qualitative, the chi-square test or Fisher's exact test were used, as appropriate. Correlations between continuous variables with the parameters of PSG were performed using the Pearson correlation test or Spearman correlation test. For the statistical analysis, we used the statistical software Statistical Package for Social Sciences 18.0® SPSS (Chicago, IL, USA). The level of statistical significance was set at 5% for all tests (*p* < 0.05).

## Results

**Fig 2:**
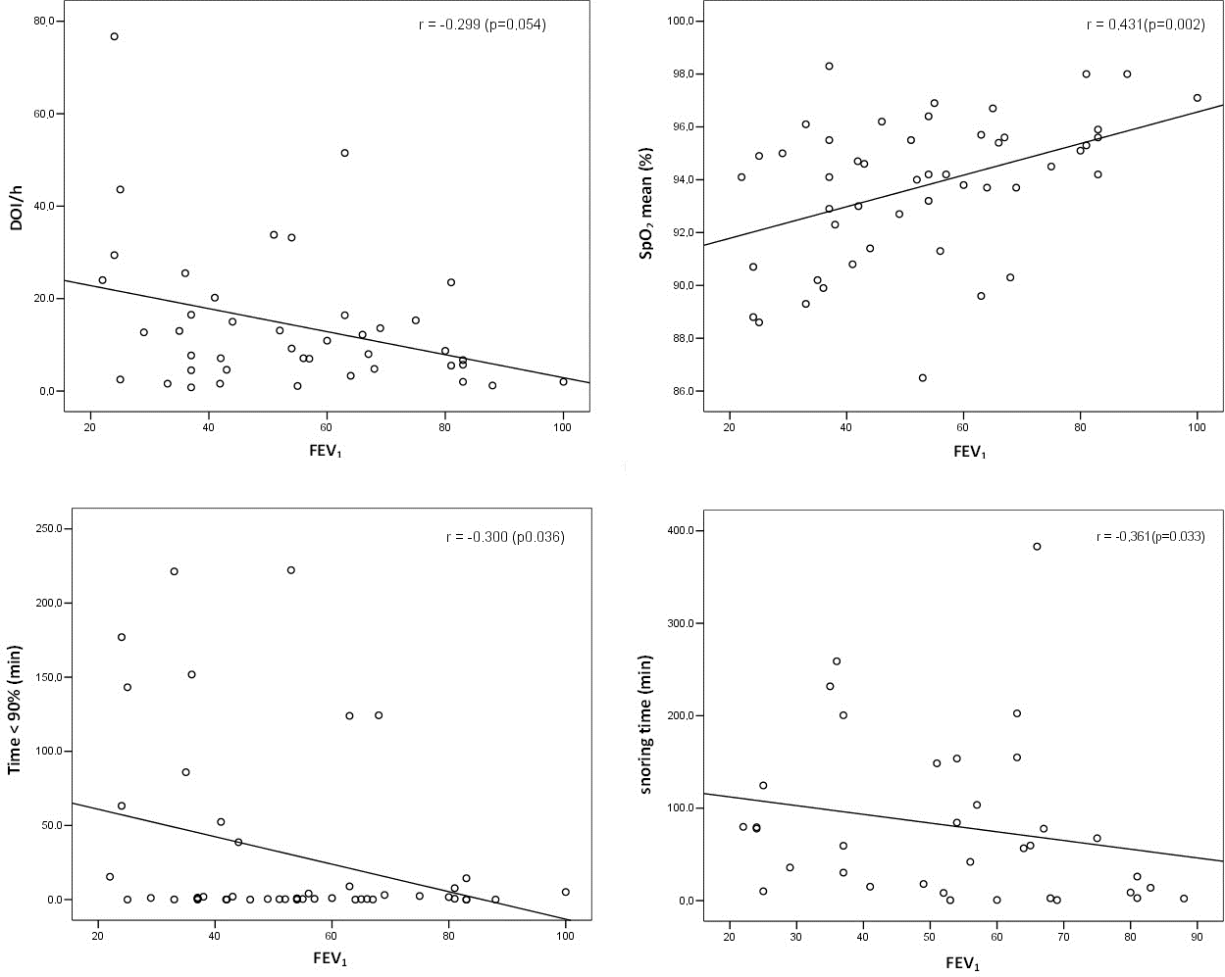
Correlation between FEV_1_ and sleep parameters. FEV_1_: forced expiratory volume in the first second; h: hours; min: minutes; ODI: oxygen desaturation index; OSA: obstructive sleep apnea; SD: standard deviation; SpO_2_mean: mean peripheral capillary oxygen saturation.

The final sample in this study involved 49 patients. The clinical, demographic, and anthropometric characteristics and comorbidities of all enrolled patients are shown in Table 1. The majority of NCFB cases (51%) were attributed to post-infection origin. Of the 47 patients (2 patients without a medical record documenting infection), 9 (18.4%) had PA colonization at baseline, and 12 (24.5%) had exacerbation ≥ 3 times per year. Additionally, of the 49 total patients in the study, 27 were prescribed azithromycin (3 times per week), 6 patients were using home oxygen therapy, and one patient used benzodiazepines. The spirometric characteristics of patients diagnosed with NCFB are shown in Table 2. Obstructive breathing patterns were predominant (59.2%). FEV_1_% predicted was negatively correlated with snoring time (*r* = - 0.361, *p* = 0.033), oxygen desaturation index (ODI) per hour (*r* = - 0.299, *p* = 0.054) and total sleep time (TST) with peripheral capillary oxygen saturation (SpO_2_) < 90% (*r* = - 0.300, *p* = 0.036), as shown in Fig 2. No significant correlations were found between FEV_1_% predicted and any other sleep variables.

**Table 1:** Clinical, demographic, and anthropometric characteristics and comorbidities of all enrolled patients.

Characteristics	n = 49
Age, y	50.3 ± 13.6
Male sex, No (%)	27 (51.1)
BMI, kg/m^2^	23.8 ± 3.4
Cough	48 (97.9)
Dyspnea	39 (79.6)
Lobectomy	13 (26.5)
Hemoptysis	17 (34.7)
Rhinosinusitis	13 (26.5)
*Pseudomonas aeruginosa* colonization	9 (18.4) ^ⱡ^
Exacerbation frequency in last year	1 (1.00–8.00)
Exacerbation ≥3 times per year	12 (24.5) ^ⱡ^
Causes, No (%)	
Post-infective	25 (51.0)
Tuberculosis sequelae	19 (38.8)
Others	5 (10.2)
Comorbidities (%)	
Hypertension	17 (34.7)
Chronic cardiac disease	7 (14.3)
Cerebrovascular disease	3 (6.1)
Diabetes mellitus	2 (4.1)

BMI: body mass index; IQR: interquartile range; Kg: kilogram; m^2^: square meters; SD: standard deviation; y: years. Data are expressed as mean (±SD), median (IQR) or No. (%).

^ⱡ^ There was no information in the medical record for 2 patients.

**Table 2:** Spirometric variables of patients with non-cystic fibrosis bronchiectasis (n = 49).

	Pre-BD	Post-BD	*P* value
FEV_1,_ L	1.56 ± 0.64	1.66 ± 0.67	<0.001
FEV_1_% predicted	52.56 ± 19.35	57.39 ± 21.48	<0.001
FVC, L	2.49 ± 0.75	2.58 ± 0.75	0.010
FVC % predicted	68.01 ± 17.76	70.93 ± 17.91	0.002
FEV_1_/FVC %	68.46 ± 16.63	68.27 ± 18.17	ns

BD. Bronchodilator; FEV_1_: forced expiratory volume in the first second; FVC: forced vital capacity; L: liters; NS: no significant difference; SD: standard deviation. Data are expressed as mean (± SD) or No. (%).

PSG physiological variables, ESS scores, and Berlin Questionnaire results for all patients are shown in Table 3. The values referring to physiological sleep variables were obtained through Overnight PSG recordings and compared to normal values for the adult population considered in the scientific literature [49].

**Table 3:** Polysomnographic physiological variables, Epworth Sleepiness Scale score and Berlin Questionnarie results in patients with non-cystic fibrosis bronchiectasis (n = 49).

Variables	Values	Reference values
TST (min)	325.15 ± 64.22	300–360
Sleep Efficiency (%)	84.01 ± 14.16	> 85%
Sleep Stadiums		
E1 (%TST)	9.97 ± 8.45	3–5
E2 (%TST)	44.72 ± 12.11	45–50
E3(%TST)	22.65 ± 15.08	18–20
REM (%TST)	17.33 ± 9.05	20–25
Snoring presence (%)	71.43	-
Snoring time (TST) (min)	80.63 ± 89.02	-
Arousals index (events/h)	11.02 ± 10.76	> 15
Arousals presence, (%)	14.29	-
AHI (events/h)	7.63 ± 11.53	AHI < 5
AHI, severity of OSA		
Mild (%)	24.49	AHI 5–15
Moderate (%)	12.24	AHI 16–30
Severe (%)	4.08	AHI > 30
ODI/h	14.35 ± 15.36	-
SpO_2_mean (%)	93.76 ± 2.71	> 90%
SpO_2_nadir (%)	83.29 ± 7.99	> 90%
TST<SpO_2_ 90% (min)	30.21 ± 60.48	-
ESS score	10.65 ± 5.97	< 10
Excessive daytime sleepiness (ESS≥ 10) (%)	26 (53.06)	-
Berlin Questionnaire (high risk for OSA) (%)	27 (55.10)	Low risk

Data are expressed as mean (± SD), or No. (%). AHI: apnea-hypopnea index; ESS: Epworth sleepiness scale; E1: sleep stage 1; E2: sleep stage 2; E3: sleep stage 3; h: hours; min: minutes; ODI: oxyhemoglobin desaturation index; OSA: obstructive sleep apnea; REM: rapid eyes movement; SD: standard deviation; SpO_2_mean: mean oxyhemoglobin saturation; SpO_2_nadir: minimum oxyhemoglobin saturation; TST: total sleep time; EDS: Excessive daytime sleepiness.

The 49 patients were divided into 2 groups according to the presence or absence of OSA. Comparison of both groups (OSA vs. non-OSA) demonstrated significant differences in NC, SpO_2_ nadir nocturnal, ODI/h, TST with SpO_2_ < 90%, PA colonization, and high risk of developing OSA (Table 4).

**Table 4:** Comparison of clinical characteristics of OSA and no-OSA non-cystic fibrosis bronchiectasis patients.

Characteristics	OSA (n = 20)	no-OSA (n = 29)	*P* value
Age (y)	50.55 ± 14.49	50.10 ± 13.22	ns
Male sex, %	80.0	37.9	0.004
BMI (kg/m^2^)	24.56 ± 2.30	22.51 ± 3.86	ns
Neck circumference (cm)	37.80 ± 2.86	34.95 ± 3.30	0.026
VEF_1_% predicted	49.15 ± 19.39	55.89 ± 19.56	ns
SpO_2_ mean nocturnal	93.02 ± 2.65	94.28 ± 2.68	ns
SpO_2_ nadir nocturnal	84 (52.0–88.0)	87 (72–95)	0.002
ODI/ h	16.4 (4.5–76.7)	6.2 (0.8–20.2)	0.001
TST < SpO_2_ 90% (min)	5.4 (0.1–177.0)	0.4 (0–222.2)	0.005
*Pseudomonas aeruginosa*	7 (35.0)	2 (7.4)ⱡ	0.026
ESS score	11.07 ± 6.16	10.43 ± 5.97	ns
Exacerbation frequency in last year	1.0 (0–7)	1.0 (0–3)	ns
Exacerbation ≥3 times per year	6 (30.0)	6 (22.2)ⱡ	ns
Berlin questionnaire (high risk for OSA)	15 (75.0)	11 (37.9)	0.011

AHI: apnea-hypopnea index; ESS: Epworth sleepiness scale; FEV_1_: forced expiratory volume in the first second; h: hours; IQR: interquartile range; min: minutes; ns: no significant difference; ODI: oxyhemoglobin desaturation index; OSA: obstructive sleep apnoea; SD: standard deviation; SpO_2_mean: mean oxyhemoglobin saturation; SpO_2_nadir: minimum oxyhemoglobin saturation; TST: total sleep time; y: years. Data are expressed as mean (± SD), median (IQR) or percentile (%).

ⱡ There was no information in the medical record for 2 patients.

In the OSA group, NC, ODI/h, TST with SpO_2_ < 90%, and PA colonization were higher and SpO_2_ nadir nocturnal was lower than the corresponding values in the non-OSA group.

Patients receiving azithromycin therapy (3 times per week) had higher TST (345.10 ± 57.82 min vs. 300.66 ± 64.4 min, *p* = 0.027) and sleep efficiency (88.63 ± 11.62% vs. 78.34 ± 11.62%, *p* = 0.007). Since patients with exacerbation ≥ 3 times per year had higher snoring time (150.54 ± 115.64 min vs. 59.84 ± 65.65 min, *p* = 0.010), and they had lower SpO_2_ nadir nocturnal (79.83 ± 7.83% vs. 84.40 ± 7.94%, *p* = 0.050) and TST with SpO_2_ < 90% (12.98 ± 31.40 min vs. 67.0 ± 84.01 min, *p* = 0.010).

**Fig 3:**
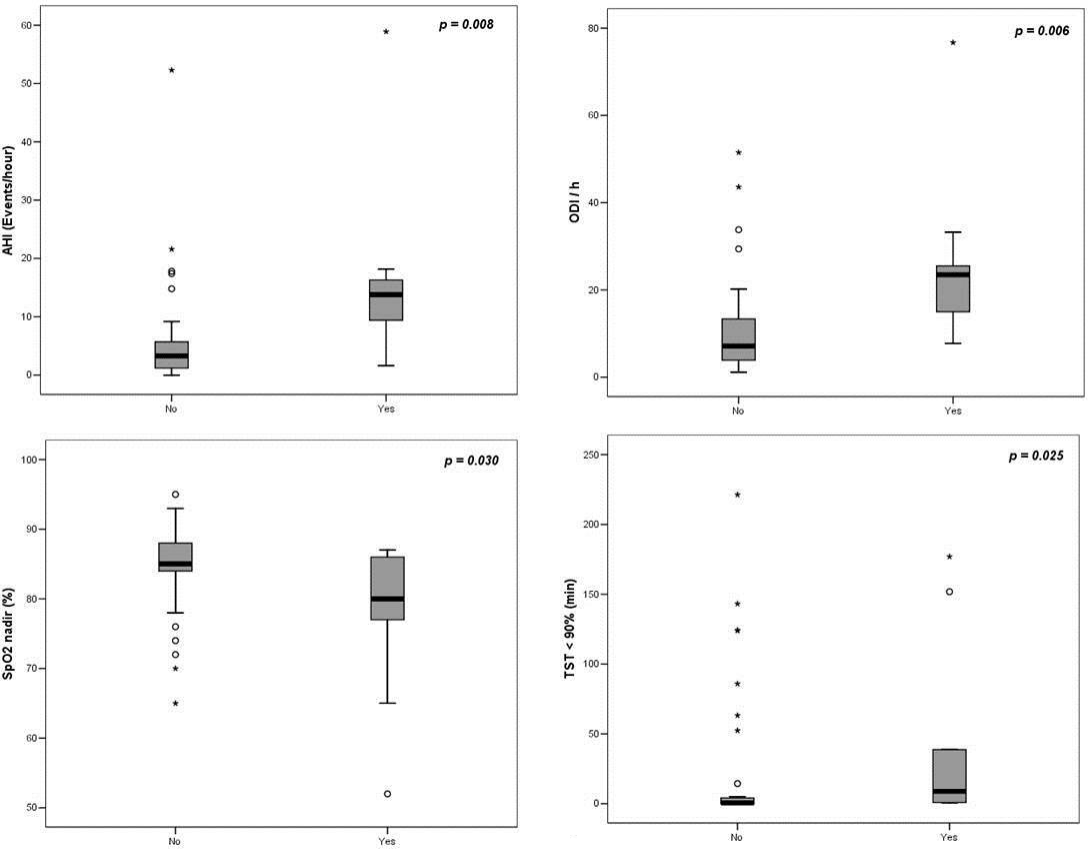
PSG parameters according to presence of *Pseudomonas aureginosa*. Each box shows median (solid black line), interquartile range (solid box), and extreme values. *p* < 0.05 was considered statistically significant.

Fig 3 shows a significant relationship between the presence of PA and AHI, ODI, and TST with SpO_2_ < 90%. The patients with NCFB with PA had higher AHI, ODI, and TST with SpO_2_ < 90%, and lower SpO_2_ nadir.

## Discussion

To our knowledge, this is the first study to describe physiological sleep variables in adult patients with NCFB, using the gold standard for evaluating sleep disorders—PSG. In our study, the prevalence of NCFB was higher in male patients; the mean BMI value demonstrates that these patients were within the ideal weight range.

The lung function of these patients with NCFB displayed predominance of obstructive lung disease, similar to that observed in the literature [50]. The majority had moderate severity, and were, on average, responsive to the use of BD. The finding of air flow limitation in the presence of bronchial dilatation can be tentatively ascribed to the fact that the dilated bronchi show inflammation, and are constantly obstructed by the presence of intraluminal secretions, with consequent reduction in lumen diameter [5].

Regarding sleep patterns, it was observed that, in general, the patients with NCFB slept less, and showed a discrete alteration in sleep efficiency and fragmented sleep architecture, with an increase in non-rapid eye movement (NREM) stage 1 sleep and reduction in rapid eye movement (REM) sleep. Young adult patients with CF exhibit sleep efficiency close to that found in this study, being 85.2% to 87.1% [28]. REM sleep is also reported to be reduced in CF patients, although the studies were performed in children [51]. The presence of snoring was also observed in 71.43% of the studied population; a higher percentage than that in patients with asthma and COPD [52–53]. Although the main symptoms in the patients with NCFB are the presence of cough and sputum, only 14.29% of the patients showed arousal—higher than that found in patients with CF [54], although lower than that recorded in a recent study involving COPD subjects [55].

However, the presence of oxygen desaturation was observed in the majority of the patients: 38.7% of the patients had an SpO_2_ nadir < 85%. The mean ODI was 14.35 ± 15.36/h and the average SpO_2_ nadir was 83.29 ± 7.99%, the mean TST with SpO_2_ < 90% being 30.21 ± 60.48 min, representing around 9% of the TST. Milross et al. [31] found, in patients with CF, an average SpO_2_ nadir nocturnal of 82.5%, which is corroborated by the current study.

The presence of cough and accumulation of secretion would result in hyperventilation, ventilatory disorders, and gas exchange alterations, contributing to hypoxemia, which occurs in patients with COPD [56]. When we analyzed the presence of OSA in the phases of REM sleep (5.7 ± 8.3) and NREM sleep (3.6 ± 9.3) and the presence of hypopnea in the REM sleep (9 ± 10.8) and NREM sleep (2.8 ± 3.9) we can observe the presence of both events, apnea and hypopnea in the two phases of sleep. This finding justifies the presence of significant SpO2 nadir, ODI, and TST with SpO2 < 90% values when comparing OSA and no-OSA patients, reinforcing the deleterious effect of OSA in this population. The fact that these variables present a significant difference when compared to OSA and no-OSA patients demonstrates the effect of obstructive events on nocturnal oxyhemoglobin saturation and not only the effect due to structural lung disease.

Another possible explanation for this reduction in SpO2 is the presence of OSA in this population, as described in this study and detailed in Table 4: there are statistically significant differences in SpO2 nadir, ODI, and TST with SpO2 < 90% depending on whether there is OSA or not. This finding is very important because Onen et al. [57] correlated hypoxemia with mortality. Another possible explanation for this reduction in SpO_2_ is the presence of OSA in this population, as described in this study and detailed in Table 4: there are statistically significant differences in SpO_2_ nadir, ODI, and TST with SpO_2_ < 90% depending on whether there is OSA or not. This finding is very important because Onen et al. [57] correlated hypoxemia with mortality.

Through assessing the ESS, it was found that 53.1% of the patients reported EDS, which is a higher percentage than that found in the general population [58], in adults with CF [59], COPD [27], and in a recent study with stable adult patients with BCTS [33], in which 31.9% exhibited EDS. It is noteworthy that the FEV_1_% predicted was lower in our study and that patients with NCFB showed a higher EDS risk, associated with the presence of cough and sputum [60], and were more commonly found in those with OSA.

As in a recent study performed with asthma patients [26], a high risk of OSA was observed in our study on NCFB. The concordance between the prevalence of high risk of OSA, assessed through the Berlin Questionnaire, and the prevalence of OSA, assessed through PSG, was confirmed by the significant difference found between the groups with and without OSA.

Katz et al [61] reported in their study that around 50% of patients with CF showed sleep disorders. In patients with BCTS, Erdem et al. [32] described 37% of children with CF exhibiting sleep disorders, while Gao et al [33] reported that 56.9% of stable adults with BCTS also demonstrated sleep disorders. Both did not directly assess the presence of OSA: they used very specific sleep questionnaires. In our study, utilizing PSG, the presence of OSA in clinically stable adult patients with NCFB was 40.28% above that encountered in the general population [20–21] and in other respiratory diseases [23, 25, 26]. Only one recent study, in patients with COPD, performed in Singapore, presented a higher OSA prevalence than that found in our study [55].

While comparing some risk factors for OSA, according to AASM [46], by regarding those with or without OSA, we verified the statistically significant difference regarding male gender and NC.

The neck circumference values presented a significant difference when compared to the OSA and non-OSA groups. However, when comparing the mean values obtained in the population of this study with the reference values proposed in the literature (41 cm for women and 43 cm for men), it can be observed that these are below the cut-off values for the Presence of OSA. The BMI values did not present significant difference between the two groups and when compared to the reference values proposed by the WHO it was observed that they are underweight, clinical characteristic of the disease.

When we verified the presence of rhinosinusitis in the patients involved in this study, it was observed that only 13 patients (26.5%) presented complaints. In these patients, the mean value of AHI was 5.8 events per hour. Three patients presented mild AHI, three had moderate AHI and the other patients (7 patients) presented no OSA.

Patients with chronic nasal and sinus inflammation usually have nonspecific bronchial hyperresponsiveness, suggesting a neural reflex. Postnasal drainage of nasal inflammatory mediators during sleep in patients with sinus disease may also increase lower airway responsiveness. [62] We could think that the sinus disease would impact on nasal patency and could tip the people toward higher likelihood of OSA. However, according to the data obtained in this study and according to the lack of scientific studies about a possible association, we can not consider the presence of rhinosinusitis as a risk factor for OSA.

The FEV_1_% predicted did not show a statistically significant difference depending on the presence or absence of OSA, although in the OSA group the FEV_1_ value was < 50% of predicted, which is a predictor of the severity of BCTS [63].

Another severity predictor, the presence of PA, was higher in patients with OSA (*p* = 0.026). Besides that, patients with NCFB that were colonized with PA showed higher AHI and greater oxygenation changes, with higher ODI and TST with SpO_2_ < 90% and lower SpO_2_ minimum. Probably the mechanism entails worsening of pulmonary function and greater production of secretions, caused by colonization with this bacterium [5]. We can hypothesize that the presence of PA, by provoking systemic and airways inflammation—in addition to being responsible for the worsening of pulmonary function [64] could lead to apnea and hypopnea, given that patients with OSA normally show airways inflammation.

Additionally, patients with ≥ 3 exacerbations per year, also considered a BCTS severity predictor, exhibit longer snoring periods, worse SpO_2_ nadir and higher TST with SpO_2_ < 90%. Regarding all the patients with NCFB, 55.1% were receiving drug treatment with azithromycin (3 times a week), indicated as immunomodulatory and anti-inflammatory therapy [65–67]. In the group of patients with ≤ 3 exacerbations per year, higher TST and sleep efficiency were observed, probably due to the reduction in secretions and anti-inflammatory effects.

We believe that the results of this study draw the attention of the scientific community to the importance of research into the presence of sleep-disordered breathing in patients with BCTS and the possible indication for treatment of these patients with non-invasive ventilator support.

## Conclusion

Adult patients with clinically stable NCFB, especially those infected with PA, exhibit EDS and high prevalence of OSA, associated with considerable oxygen desaturation during sleep.

## Supporting information

S1 File Database. (XLS)
